# Macrophages’ M1 or M2 by tumor microparticles: lysosome makes decision

**DOI:** 10.1038/s41423-022-00892-z

**Published:** 2022-06-30

**Authors:** Ke Tang, Jingwei Ma, Bo Huang

**Affiliations:** 1 Department of Biochemistry and Molecular Biology, Tongji Medical College, Huazhong University of Science and Technology, 430030 Wuhan, China; 2 Cell Architecture Research Center, Huazhong University of Science and Technology, 430030 Wuhan, China; 3 Department of Immunology, Tongji Medical College, Huazhong University of Science and Technology, 430030 Wuhan, China; 4 Department of Immunology & National Key Laboratory of Medical Molecular Biology, Institute of Basic Medical Sciences, Chinese Academy of Medical Sciences (CAMS) & Peking Union Medical College, 100005 Beijing, China

**Keywords:** Immunology, Tumour immunology

Cells deliver messages to their surroundings or distant cells by secreting signaling molecules or releasing extracellular microvesicles (EVs) [1, 2]. Two types of EVs have been identified: exosomes and microparticles (MPs), which are also termed microvesicles (MVs). Exosomes are endosome-derived EVs (30–100 nm) that deliver information about proteins, mRNAs, and microRNAs to recipient cells [3]. MPs are plasma membrane-derived shedding vesicles with sizes ranging from 0.1 to 1 μm [4]. Relative to the substantial study of exosomes, knowledge of MPs is severely lacking, especially in cancer research. Due to the chaotic nature of the tumor microenvironment, tumor cells release a large number of MPs (tumor cell-derived MPs or T-MPs). Despite their impact on tumor cells themselves, T-MPs can profoundly influence tumor-infiltrating immune cells, especially macrophages, due to their highly efficient uptake of T-MPs.

As professional phagocytes, macrophages have a remarkable ability to engulf extracellular particles. In an in vitro coculture assay, T-MPs were observed in macrophages within <2 min. These phagocytosed T-MPs, however, effectively polarized the macrophages toward a typical M2 phenotype, leading to tumor immunosuppression and promoting tumor growth [5]. In another study, circulating T-MPs were found to enter alveolar sites, where they were taken up by alveolar macrophages and induced them to produce CCL2, thus inducing chemoattraction of inflammatory monocytes into the lung parenchyma and promoting the formation of a premetastatic niche by reprogramming the inflammatory and mechanical microenvironment [6]. These findings imply potential roles for T-MPs in cancer treatment. In EGFR mutation-driven lung cancer, treatment with tyrosine kinase inhibitors induces tumor cell apoptosis; meanwhile, abundant T-MPs are released into the tumor microenvironment. These T-MPs contain long noncoding RNAs from treated tumor cells, which are delivered to macrophages by the T-MPs, resulting in the release of the proinflammatory cytokine IL-1β [7]. Thus, T-MPs may not only mediate an immunosuppressive phenotype but also induce a proinflammatory or even a mixed phenotype of tumor-associated macrophages dependent on the messenger molecules they carry. How T-MPs reprogram the polarization of macrophages remains to be determined.

Previous mechanistic studies have shown that T-MPs activate the cGAS-STING-TBK1-STAT6 axis and induce the expression of anti-inflammatory molecules in macrophages [5]. This effect is attributable to DNA signals, since both genomic and mitochondrial DNA fragments can be contained in T-MPs. However, when these T-MPs are taken up by dendritic cells (DCs), they also triggering activation of the cGAS-STING-TBK1 axis, resulting in an immune activation phenotype with the upregulation of CD80, CD86 and MHCII. This occurs because activated TBK1 targets IRF3/IRF7 and induces type I interferon expression in DCs. How do the same T-MPs result in different consequences in macrophages and DCs? The answer might lie in lysosomes. Following the uptake of T-MPs, the lysosomal pH decreases in macrophages; in contrast, the lysosomal pH increases in DCs.

Phagocytes perform phagocytosis to take up extracellular virions or cellular particles, leading to the formation of endosomes in the cytoplasm; here, the endosomes are processed through early and late stages via luminal acidification and fuse with lysosomes for cargo degradation [8]. Normally, the lysosomal pH value ranges from 4.5 to 5.0, mainly achieved by vacuolar H^+^-ATPases, which is located in lysosomal membranes and pumps cytosolic H^+^ into the lysosomal lumen [9]. Strikingly, T-MPs can elevate the lysosomal pH to as high as 8.0. How does this happen? T-MPs are able to activate the lysosomal NOX2 (also called gp91^phox^) NADPH oxidase, leading to the production of superoxide anions, which consume H^+^ to yield hydrogen peroxide in DCs [10]. Furthermore, increased lysosomal ROS can activate Ca^2+^ release from lysosomes, rather than the ER or mitochondria, into the cytosol, where activated Ca^2+^ signaling promotes the mRNA expression and nuclear translocation of transcription factor EB (TFEB), a master regulator of autophagy and cellular metabolism, thus facilitating a proinflammatory phenotype of DCs. This prompted us to wonder whether T-MPs can be used to deliver certain drug(s) that can induce M2 macrophage polarization into M1 macrophages by modifying lysosomal pH and ROS levels.

A typical trait of MPs lies in their carrier function. Chemotherapeutic drugs can be packaged by T-MPs for cancer treatment [11]. Once drug-carrying MPs gain access to the tumor microenvironment, TAMs readily take up the MPs; in turn, drug molecules are delivered by T-MPs to the lysosomes of TAMs. Cells have evolved the cytochrome P450 system to detoxify toxins, including chemotherapeutic drugs [12]. As heme-containing monooxygenases, P450 enzymes can induce the transfer of NADPH-donated electrons to molecular oxygen to generate O^2−^ and the subsequent formation of H_2_O_2_. Notably, P450 enzymes not only exist in the cytosol but also are present in lysosomes, where lysosomal drugs can be metabolized. This process may facilitate the generation of P450-driven lysosomal ROS, which subsequently trigger the activation of the lysosomal NOX2 system that can amplify ROS production. As a result, lysosomal pH is increased, and Ca^2+^ ions are released. Therefore, such molecular events confer drug-packaging T-MPs the ability to polarize M2 macrophages into M1 macrophages.

In summary, the lysosome not only is a place for cargo degradation but also plays crucial roles in regulating macrophage polarization. T-MPs, by virtue of their entry into lysosomes, can polarize tumor-associated macrophages into an M2 phenotype through the lysosome-dependent pathway. Loaded chemotherapy drugs, however, are able to polarize M2 macrophages into M1 macrophages by modulating lysosomal ROS, pH and Ca^2+^ release. The paramount importance of lysosomes in macrophage polarization is just beginning to be appreciated. Future studies in this field will undoubtedly strengthen our understanding of the biology and pathophysiology of macrophages.
